# Aurora-A Identifies Early Recurrence and Poor Prognosis and Promises a Potential Therapeutic Target in Triple Negative Breast Cancer

**DOI:** 10.1371/journal.pone.0056919

**Published:** 2013-02-20

**Authors:** Jie Xu, Xing Wu, Wei-hua Zhou, An-wen Liu, Jian-bing Wu, Jin-yun Deng, Cai-feng Yue, Shao-bing Yang, Jing Wang, Zhong-yu Yuan, Quentin Liu

**Affiliations:** 1 State Key Laboratory of Oncology in South China, Cancer Center, Sun Yat-sen University, Guangzhou, China; 2 Department of Oncology, The Second Affiliated Hospital, Nanchang University, Nanchang, China; 3 Department of Hematology, the Third Affiliated Hospital, Sun Yat-sen University, Guangzhou, China; 4 Department of the Third Internal Medicine, Jiangxi Province Cancer Hospital, Nanchang, China; 5 Department of Anesthesiology, Tongji Hospital of Tongji Medical College, Huazhong University of Science and Technology, Wuhan, China; 6 Department of Medical Oncology, Cancer Center, Sun Yat-sen University, Guangzhou, China; 7 Institute of Cancer Stem Cell, Dalian Medical University, Dalian, China; Cedars-Sinai Medical Center, United States of America

## Abstract

Triple negative breast cancer (TNBC) acquires an unfavorable prognosis, emerging as a major challenge for the treatment of breast cancer. In the present study, 122 TNBC patients were subjected to analysis of Aurora-A (Aur-A) expression and survival prognosis. We found that Aur-A high expression was positively associated with initial clinical stage (*P* = 0.025), the proliferation marker Ki-67 (*P* = 0.001), and the recurrence rate of TNBC patients (*P*<0.001). In TNBC patients with Aur-A high expression, the risk of distant recurrence peaked at the first 3 years and declined rapidly thereafter, whereas patients with Aur-A low expression showed a relatively constant risk of recurrence during the entire follow-up period. Univariate and multivariate analysis showed that overexpression of Aur-A predicted poor overall survival (*P* = 0.002) and progression-free survival (*P* = 0.012) in TNBC. Furthermore, overexpression of Aur-A, associated with high Ki-67, predicted an inferior prognosis compared with low expression of both Aur-A and Ki-67. Importantly, we further found that Aur-A was overexpressed in TNBC cells, and inhibition of this kinase inhibited cell proliferation and prevented cell migration in TNBC. Our findings demonstrated that Aur-A was a potential therapeutic target for TNBC and inhibition of Aur-A kinase was a promising regimen for TNBC cancer therapy.

## Introduction

Breast cancer is one of the leading causes of cancer-related death among adult females in the world [1]. The management and prognosis of breast cancer are largely determined by the expression status of estrogen receptor (ER), progesterone receptor (PR), and human epidermal growth factor receptor 2 (HER2) of the tumor [2]–[4]. The hormone receptor (HR) – expressing tumors benefit from endocrine therapy and generally carry a favorable prognosis [2]–[4]. Combination of trastuzumab with adjuvant chemotherapy in HER2/neu-positive patients also significantly improve the outcome of these patients [5]. However, TNBC, defined as a tumor subtype that lack of ER, PR and HER2 expression, shows a poor prognosis due to its aggressive tumor biology and resistance to most available endocrine and molecularly targeted treatments [6]–[8]. Accordingly, TNBC presents as a major challenge for the development of effective therapeutic strategies of breast cancer [9]. Previous reports showed that TNBC was associated with a high expression of the proliferation biomarker Ki-67 [10], as well as a few molecular targets including vascular endothelial growth factor (VEGF) [11], and epidermal growth factor receptor (EGFR) [12]–[14]. Identification and characterization of more novel molecular biomarkers, which may also act as potential therapeutic targets in TNBC, are urgently needed.

Aur-A (also known as STK15, BTAK, and Aurora 2), as a member of mitotic serine/threonine Aurora kinase family, is essential in accurate timing of mitosis and maintenance of bipolar spindles [15]–[16]. Overexpression of Aur-A in mouse mammary epithelium resulted in genetic instability and subsequent tumor formation [17]. Inhibition of Aurora kinases suppressed tumor growth in vivo [18]. Furthermore, Aur-A was overexpressed or amplified in hepatocellular carcinoma [19], laryngeal squamous cell carcinoma (LSCC) [20], esophageal squamous cell carcinoma [21], ovarian cancer [22], and neuroblastoma [23], contributing to tumor progression and poor prognosis. Thus, inhibition of Aur-A kinase is a promising regimen for cancer therapy [20], [23].

In breast cancer, Aur-A expression was found to be elevated at protein and mRNA levels in the tumor specimens [24], [25]. In addition, high expression of Aur-A rather than Aur-B predicts a decreased survival time in breast cancer [26]. Recently, we found that overexpression of Aur-A enhanced cell migration and promoted breast cancer metastasis by dephosphorylating and activating cofilin [27]. This event could be suppressed markedly by the small molecule inhibitor of Aur-A kinase [27]. Thus, Aur-A promised a potential therapeutic target in breast cancer. However, the precise role of Aur-A in TNBC is still unknown, and there is little study focusing on the clinical significance of Aur-A in TNBC.

The purpose of this study was to detect the protein expression of Aur-A in TNBC patients and analyze its clinicopathological/prognostic significances in this tumor subtype. Our results showed that high expression of Aur-A in TNBC was positively associated with malignant pathological features and predicted adverse overall survival (OS) and progression-free survival (PFS). Moreover, we found that inhibition of Aur-A could inhibit cell proliferation and reduce cell migration in TNBC cells, emerging as a potential targeted treatment for TNBC.

## Materials and Methods

### Patients

Between January 1997 and October 2003, a total of 628 female breast cancer patients who received postoperative adjuvant chemotherapy (Cyclophosphamide/Adriamycin/5-Fluorouracil (CAF) or Cyclophosphamide/Methotrexate/5-Fluorouracil (CMF)) at Cancer Center of Sun Yat-sen University (Guangzhou, China) were included in the present study. Among the 628 breast cancer patients, 149 patients were classified as TNBC. Staging was performed according to American Joint Committee on Cancer guidelines [28]. Of the overall TNBC patients (n = 149), no patients received preoperative chemotherapy and radiotherapy. All the TNBC patients (n = 149) received mastectomy surgery (no patients received breast conserving surgery), and none was identified as inflammatory breast cancer. Of the 149 eligible women identified with triple-negative breast tumors, 17 patients with loss of follow-up and 10 patients with lack of adequate tissues were excluded from this study, leaving 122 patients subjecting to further clinical and survival analysis. In the cohort of 122 TNBC patients (median age, 47.0 year; range, 22–74 year), 46 patients were censused as death due to tumor progression during the 8 years of follow-up time. Written informed consent for the use of the tissues was obtained from all patients before surgery, and the study was approved by the Institute Research Ethics Committee of Sun Yat-sen University.

### Cell Lines and Cell Culture Condition

Human breast epithelial cell lines (MDA-MB-231, MDA-MB-468, BT549, MCF-7, ZR75-1, BT474, MDA-MB-435, and SKBR3) were obtained from the American Type Culture Collection (ATCC). MDA-MB-231, MCF-7, SKBR3 and MDA-MB-435 were routinely maintained in high-glucose DMEM (Gibco, C11995). BT549, ZR75-1, MDA-MB-468 and BT474 were routinely maintained in RPMI1640 (Gibco, C11875). All media were supplemented with 10% fetal bovine serum (Hyclone, SV30087.02), penicillin (100 units/mL; Sigma, P3032), and streptomycin (100 units/mL; Sigma, S9137). All cells were cultured at 37°C in humidified 5% CO_2_ incubator.

### Tissue Microarray Construction

The tissue microarrays (TMAs) were constructed as a method described previously [29]. Briefly, the hematoxylin and eosin–stained (H&E) slides were reviewed by a senior pathologist (ZY Yuan) to determine and mark the representative tumor areas. The paraffin-embedded tissue blocks and the corresponding H&E slides were then overlaid for TMA sampling. Triplicate cylindrical tissue samples with 0.6-mm in diameter were punched from individual donor tissue block (duplicated cylinders from representative cancer areas and one cylinder from adjacent normal tissues). The tissue cylinders were then transferred into a recipient paraffin block at defined positions by using a tissue-arraying instrument (Beecher Instruments, Silver Spring, MD, USA). Subsequently, multiple sections (5 µm thick) were cut from the TMA blocks and mounted on the microscope slides. One section from the TMA block was used to be stained with H&E to confirm that the punches contained tumor.

### Immunohistochemistry Assay

The immunohistochemistry (IHC) assay was performed as previously described [30]. The TMAs slides were deparaffinized in xylene, rehydrated through graded alcohol, and subsequently immersed in 3% hydrogen peroxide for 10 min to block endogenous peroxidase activity. An antigen retrieval process was accomplished by pressure cooking for 3 to 5 min. Then the slides were incubated with the primary antibody of Aur-A (monoclonal mouse; 1∶100; Sigma, A1231), ER (monoclonal rabbit; 1∶100; Thermo, SP1), PR (monoclonal mouse; 1∶100; Dako, PgR 636), HER2 (monoclonal rabbit; 1∶100; Cell Signal, #2242), and Ki67 (monoclonal mouse; 1∶150; Dako, MIB-1), overnight at 4°C, respectively. After being incubated with the secondary antibody (Rabbit/Mouse, Dako REAL™ EnVision™ Detection System, K500711) for 30 min at room temperature, specimens were stained with DAB (3, 3-diaminobenzidine; Dako REAL™ EnVision™ Detection System, Peroxidase/DAB+, K500711). Finally, the sections were counterstained with hematoxylin, dehydrated and mounted. Negative controls were employed by replacing the primary antibody with non-immune serum immunoglobulins. Known immunostaining-positive slides were used as positive controls.

The staining intensity and extent of Aur-A was graded as described previously, and the merged overall score >5 was regarded as high staining [27], [31]. ER and PR were defined as positive when ≥10% tumor cells were nuclear positively stained [10], and HER2 was defined as positive when scored as 3+ by IHC [32]. Ki-67 was considered as high expression if there was >10% positive average nuclear staining of any intensity as previously reported [33]. TNBC subtype was defined as ER (−), PR (−), and HER2 (−). Immunohistochemical staining was assessed and scored by two independent pathologists (Drs. X Wu and WH Zhou) who were blinded to the clinicopathological data. Their conclusions were in complete agreement in 85% (104/122) for Aur-A, 100% (122/122) for ER, PR, and HER2, and 88% (107/122) for Ki67, suggesting that the scoring systems were highly reproducible. The interobserver disagreements were reviewed for a second time, followed by a conclusive judgment by both pathologists.

### 3-(4, 5-Dimethylthiazol-2-yl)-2, 5-diphenyltetrazolium Bromide (MTT) Assay

MTT (Sigma, M2003) assay was used to assess the growth of MDA-MB-231, MCF-7, MDA-MB-468, and MDA-MB-435 cells. The cells under indicating treatments were plated in 96-well plates and culture for 24 h. Cell survival was assessed as described previously [34]. The experiment was repeated in the same condition for at least three times and standard deviation (SD) was determined.

### Transwell Migration Assay

Transwell assay was performed as described previously [20]. In brief, cells were added to the top chambers of 24-well transwell plates (Corning, Inc.), which were pretreated with 1% Matrigel (BD Biosciences) in PBS. Different treated cells in the top chambers were then incubated in serum-free media for 24 h. After incubation, top cells were removed and bottom cells were fixed and stained with 4, 6-diamidino-2-phenylindole (5 µg/mL) to visualize nuclei. The number of migrating cells in five fields was counted under fluorescence microscope, and the average of each chamber was determined.

### Western Blot Analysis

Western blot analysis was performed as described previously [35]. Briefly, equal amounts of cell extract were subjected to electrophoresis in SDS-polyacrylamide gel and transferred to nitrocellulose membrane (Bio-Rad Laboratories, 162-0094) for antibody blotting. The membrane was then blocked, and incubated with mouse anti-glyceraldehyde 3-phosphate dehydrogenase (GAPDH) antibody (Abmart, M20028), rabbit anti-thr288 p-Aur-A antibody (Sigma, SAB4300270), and mouse anti-Aur-A antibody (Sigma, A1231) overnight at 4°C.

### RNAi Treatment

The siRNA oligonucleotides targeting to Aur-A (si1: sense 5′AUGCCCUGUCUU ACUGUCATT3′ anti-sense 5′UGACAGUAAGACAGGGCAUTT3′, si2: sense 5′GGCAACCAGUGUACCUCAUTT3′ anti-sense 5′AUGUGGUACACUGGUUG CCTT3′, si3: sense 5′AUUCUUCCCAGCGCGUUCCTT3′ anti-sense 5′GGAACG CGCUGGGAAGAAUTT3′) and control (sense 5′UUCUCCGAACGUGUCACGU TT3′ anti-sense 5′ACGUGACACGUUCGGAGAATT3′) were transfected into tumor cells (MDA-MB-231, MCF-7, MDA-MB-468, MDA-MB-435) for 48 h using Lipofectamine 2000 (Invitrogen Life Technologies, 11668-019) according to manufacturer’s instructions. Downregulation of the proteins were demonstrated by Western blot.

### Follow-up

All TNBC patients (n = 122) had a follow-up record for over 8 years. After the completion of therapy, patients were observed at 3 month intervals during the first 3 years and at 6 month intervals thereafter. The latest follow-up was updated in September 2011. OS was defined as the time from the date of definitive surgery to the date of last follow-up or death; PFS was defined as the time from the date of definitive surgery to the date of last follow-up or disease relapse [36]. The relapse of breast cancer was defined as local failure or distant recurrence/metastasis.

### Statistical Analysis

Statistical analysis was performed using SPSS V. 17.0 (SPSS, Inc, Chicago, IL). The *χ^2^* test and student *t* test were used to make statistical comparisons between groups. The effect of predictive variables was evaluated by Cox univariate and multivariate regression models. The relationships between Aur-A expression and OS, PFS were determined by Kaplan-Meier analysis. The log-rank tests were performed to value the difference in survival probabilities between patient subsets. All *P* values quoted were two-sided and *P*<0.05 was considered statistically significant.

## Results

### Aur-A Expression and Clinical Features

**Figure 1:**
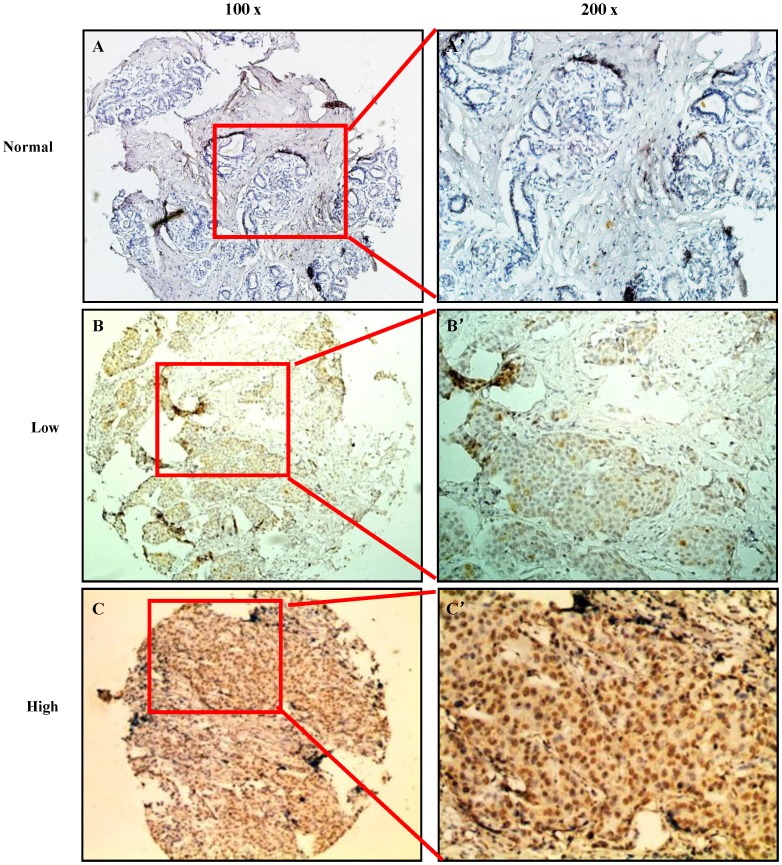
Immunohistochemistry analysis of Aur-A expression in normal and TNBC tissues. (A) Normal breast tissue showed nearly negative expression of Aur-A (100×). (B) Low expression of Aur-A was shown in a TNBC patient sample (100×). (C) Overexpression of Aur-A was detected in another TNBC case (100×). (A ’), (B ’), (C ’) demonstrated the higher magnification (200×) from the area of the box in (A), (B), (C), respectively.

Clinical features of the 122 TNBC patients, including age, family history, pathological characteristics, lymph node status, initial clinical stage, tumor stage, Ki-67, adjuvant radiotherapy, adjuvant chemotherapy, and recurrence, were summarized in Table 1. Immunoreactivity of Aur-A was observed primarily in the cytoplasm, with occasionally yellowish brown granules seen in the nuclei (Figure 1, Figure S1), whereas the proliferation marker Ki-67 was mainly expressed in the nuclei (Figure S2). In the cohort of 122 TNBC patients, high expression of Aur-A was examined in 63 of 122 (51.6%) patients and low expression of Aur-A was examined in 59 of 122 (48.4%) patients.

**Table 1:** Association of Aur-A expression with patient’s clinicopathologic characteristics in TNBC (n = 122).

Variable	All cases	Aur-A		
		High expression	Low expression	*P*
**Age (Years)**				
≥47.00	**59**	**32**	**27**	**0.578**
<47.00	**63**	**31**	**32**	
**Family history**				
Yes	**16**	**5**	**11**	**0.080**
No	**106**	**58**	**48**	
**Pathologic characteristics**				
Invasive ductal carcinoma	**117**	**60**	**57**	**1.000**
Others	**5**	**3**	**2**	
**Lymph node status**				
Negative	**44**	**17**	**27**	**0.031**
Positive	**78**	**46**	**32**	
**Initial clinical stage**				
I	**8**	**2**	**6**	**0.025**
II	**67**	**30**	**37**	
III	**47**	**31**	**16**	
**Tumor stage**				
T_1_+T_2_	**100**	**53**	**47**	**0.521**
T_3_+T_4_	**22**	**10**	**12**	
**Ki-67**				
Low	**71**	**28**	**43**	**0.001**
High	**51**	**35**	**16**	
**Adjuvant radiotherapy**				
Yes	**39**	**25**	**14**	**0.059**
No	**83**	**38**	**45**	
**Adjuvant chemotherapy**				
Yes	**115**	**60**	**55**	**0.711**
No	**7**	**3**	**4**	
**Recurrence**				
Local	25	10	15	**0.000**
Distant	42	32	10	
No	**55**	**21**	**34**	

Our data showed that Aur-A high expression was positively correlated with initial clinical stage (*P* = 0.025, Table 1), Ki-67 (*P* = 0.001, Table 1), and the recurrence rate of TNBC patients (*P*<0.001, Table 1). We further found that TNBC patients with Aur-A high expression showed a significantly high recurrence rate within the first 3 years of follow-up (30/63, 47.6%; Table 2), and the risk of recurrence dropped quickly thereafter (10/63, 15.9% during 3 to 5 years of follow-up time; and 2/63, 3.2% during 5 to 8 years of follow-up time; Table 2). TNBC patients with Aur-A low expression seemed to show a relatively steady risk of recurrence throughout the entire follow-up period: 12/59, 20.3% at the first 3 years; 7/59, 11.9% during 3 to 5 years of follow-up time; and 6/59, 10.2% during 5 to 8 years of follow-up time (Table 2).

**Table 2:** The 3-, 5- and 8-year estimates for recurrence in TNBC.

Aur-A	No. of recurrence patients during 0∼3 years	%	*p*	No. of recurrence patients during 3∼5 years	%	*P*	No. of recurrence patients during 5∼8 years	%	*P*
Low (59)	12/59	20.3%	0.002	7/59	11.9%	0.523	6/59	10.2%	0.154
High (63)	30/63	47.6%		10/63	15.9%		2/63	3.2%	
Total (122)	42/122	34.4%		17/122	13.9%		8/122	6.6%	

### Aur-A Expression and Survival Analysis

**Figure 2:**
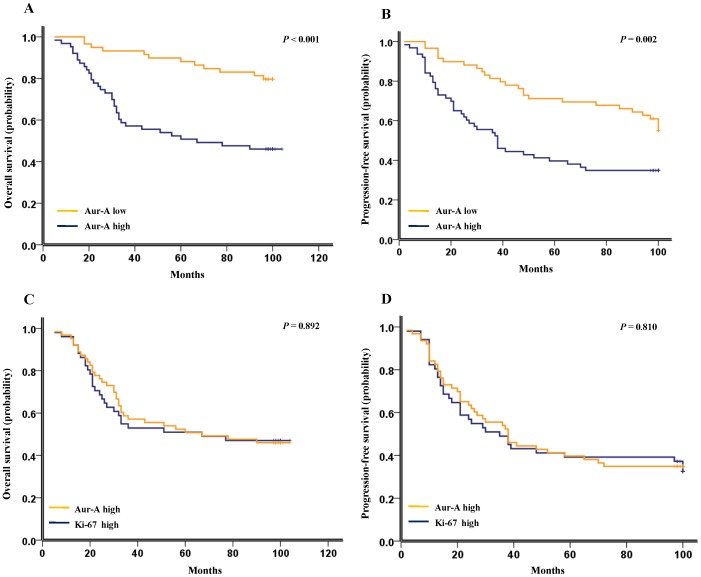
Kaplan-Meier survival analysis of Aur-A and Ki-67 expression in TNBC patients (n = 122). (A) High expression of Aur-A was closely correlated with inferior overall survival, and (B) progression-free survival in TNBC patients. The median survival time for patients with high and low expression of Aur-A was 67.5 months VS. 110.0 months for OS (*P*<0.001), and 38.4 months VS. 100.0 months for PFS (*P* = 0.002), respectively. (C) TNBC patients with high expression of Ki-67 showed similar OS and (D) PFS with those showing high Aur-A expression. The median survival time for patients with high Ki-67 expression and high Aur-A expression was 67 months VS. 67 months for OS (*P* = 0.892), and 36 months VS. 38 months for PFS (*P* = 0.810), respectively.

Our results showed that patients with Aur-A high expression had a significantly inferior OS than those with Aur-A low expression (median survival time: 67.5 months VS. 110.0 months, *P*<0.001, Figure 2A; hazard ratio, 3.631; 95% CI, 1.876–7.027; *P*<0.001; Table 3). The 3-, 5-, 8-year estimates for OS were 52.5%, 50.8%, 50.8% for TNBC patients with Aur-A high expression, and 95.2%, 85.7%, 71.4% for TNBC patients with Aur-A low expression, respectively. High expression of Aur-A also predicted an inferior PFS compared with Aur-A low expression (median survival time: 38.4 months VS. 100.0 months, *P* = 0.002, Figure 2B; hazard ratio, 2.194; 95% CI, 1.335–3.606; *P* = 0.002; Table 4). The 3-, 5-, 8-year estimates for PFS were 52.4%, 36.5%, 33.3% for TNBC patients with Aur-A high expression, and 79.7%, 67.8%, 57.6% for TNBC patients with Aur-A low expression, respectively.

**Table 3:** Results of univariate and multivariate Cox proportional-hazards analysis in TNBC patients for overall survival (n = 122).

Variable	For death					
	Univariate analysis			Multivariate analysis		
	Hazard Ratio	95% confidence interval	*P*	Hazard Ratio	95% confidence interval	*P*
Age (Years) <47.00 (VS. ≥47.00)	0.955	(0.534 to 1.706)	0.875	0.977	(0.522 to 1.829)	0.941
Family history Yes (VS. ≥ No)	0.934	(0.396 to 2.203)	0.875	2.206	(0.815 to 5.973)	0.119
Lymph node status Positive (VS. Negative)	1.530	(0.805 to 2.908)	0.194	1.660	(0.695 to 3.965)	0.254
Initial clinical stage						
I	0.174	(0.024 to 1.287)	0.087	0.336	(0.044 to 2.593)	0.296
II	0.414	(0.228 to 0.749)	0.004	0.531	(0.290 to 0.974)	0.041
III	1	1		1	1	
Pathologic characteristicsDuctal (VS. Other)	1.676	(0.519 to 5.405)	0.388	1.478	(0.430 to 5.085)	0.536
Tumor stage T_3_+ T_4_ (VS. T_1_+ T_2_)	1.500	(0.744 to 3.024)	0.257	1.618	(0.692 to 3.780)	0.267
Adjuvant chemotherapyYes (VS. No)	3.191	(0.440 to 23.153)	0.251	3.282	(0.421 to 25.600)	0.257
Adjuvant radiotherapy Yes (VS. No)	1.913	(1.067 to 3.431)	0.029	1.095	(0.520 to 2.305)	0.811
Ki-67 High (VS. Low)	2.776	(1.540 to 5.003)	0.001	1.935	(1.047 to 3.576)	0.035
Aur-A High (VS. Low)	3.631	(1.876 to 7.027)	0.000	2.846	(1.446 to 5.600)	0.002

**Table 4:** Results of univariate and multivariate Cox proportional-hazards analysis in TNBC patients for progression-free survival (n = 122).

Variable	For progression-free survival					
	Univariate analysis			Multivariate analysis		
	Hazard Ratio	95% confidence interval	*P*	Hazard Ratio	95% confidence interval	*P*
Age (Years) <47.00 (VS. ≥47.00)	0.876	(0.541 to 1.419)	0.592	0.924	(0.558 to 1.530)	0.758
Family history Yes (VS. ≥ No)	0.933	(0.462 to 1.884)	0.847	1.356	(0.618 to 2.973)	0.448
Lymph node status Positive (VS. Negative)	1.581	(0.928 to 2.694)	0.092	1.296	(0.647 to 2.594)	0.465
Initial clinical stage						
I	0.235	(0.056 to0.982)	0.047	0.293	(0.053 to 1.628)	0.161
II	0.505	(0.310 to 0.825)	0.006	0.600	(0.291 to 1.234)	0.165
III	1	1		1	1	
Pathologic characteristicsDuctal (VS. Other)	1.351	(0.490 to 3.722)	0.561	1.085	(0.368 to 3.203)	0.883
Tumor stage T_3_+ T_4_ (VS. T_1_+ T_2_)	1.717	(0.638 to 2.148)	0.611	1.045	(0.501 to 2.181)	0.907
Adjuvant chemotherapy Yes (VS. No)	1.623	(0.509 to 5.169)	0.413	1.468	(0.424 to 5.078)	0.544
Adjuvant radiotherapy Yes (VS. No)	1.999	(1.226 to 3.258)	0.005	1.739	(1.054 to 2.869)	0.030
Ki-67 High (VS. Low)	2.001	(1.187 to 3.105)	0.005	1.612	(0.978 to 2.656)	0.061
Aur-A High (VS. Low)	2.194	(1.335 to 3.606)	0.002	1.930	(1.158 to 3.217)	0.012

**Figure 3:**
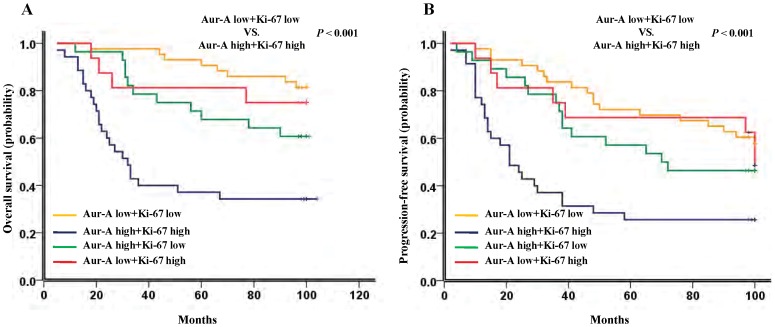
Kaplan-Meier survival analysis of Aur-A and Ki-67 expression in TNBC patients (n = 122). (A) TNBC patients with high expression of both Aur-A and Ki-67 showed an inferior overall survival (*P*<0.001), and (B) progression-free survival (*P*<0.001), compared with both Aur-A and Ki-67 low expression. The median survival time for patients with high expression of both Aur-A and Ki-67 VS. low expression of both Aur-A and Ki-67 were 32.5 months VS. 110.0 months for OS (*P*<0.001), and 21.8 months VS. 100.0 months for PFS (*P*<0.001), respectively.

Univariate analysis demonstrated that the proliferation marker Ki-67 adversely affected OS (hazard ratio, 2.776; 95% CI, 1.540–5.003; *P* = 0.001, Table 3) and PFS (hazard ratio, 2.001; 95% CI, 1.187–3.105; *P* = 0.005, Table 4) in TNBC patients. In addition, our data showed that patients with high expression of Ki-67 showed similar OS and PFS with patients with high Aur-A expression (median survival time of OS: 67 months VS. 67 months, *P* = 0.892, Figure 2C; median survival time of PFS: 36 months VS. 38 months, *P = *0.810, Figure 2D), suggesting both Aur-A and Ki-67 as similar poor prognostic factors in TNBC. Furthermore, overexpression of Aur-A, associated with high Ki-67, predicted an inferior OS (*P*<0.001, Figure 3A) and PFS (*P*<0.001, Figure 3B) compared with low expression of both Aur-A and Ki-67, indicating that Aur-A might promote tumor progression and poor prognosis through promoting cell proliferation.

Multivariate Cox analysis showed that Aur-A was a significant independent prognostic factor for OS (hazard ratio, 2.846; 95% CI, 1.446–5.600; *P* = 0.002; Table 3) and PFS (hazard ratio, 1.930; 95% CI, 1.158–3.217; *P* = 0.012; Table 4) in the cohort of 122 TNBC patients. Furthermore, Ki-67 was also found to be a significant independent prognostic factor for OS (hazard ratio, 1.935; 95% CI, 1.047–3.576; *P* = 0.035; Table 3), but not for PFS (hazard ratio, 1.612; 95% CI, 0.978–2.656; *P* = 0.061; Table 4) in TNBC patients.

### Inhibition of Aur-A Kinase Activity Inhibited Cell Proliferation and Decreased Cell Migration in TNBC Cells

**Figure 4:**
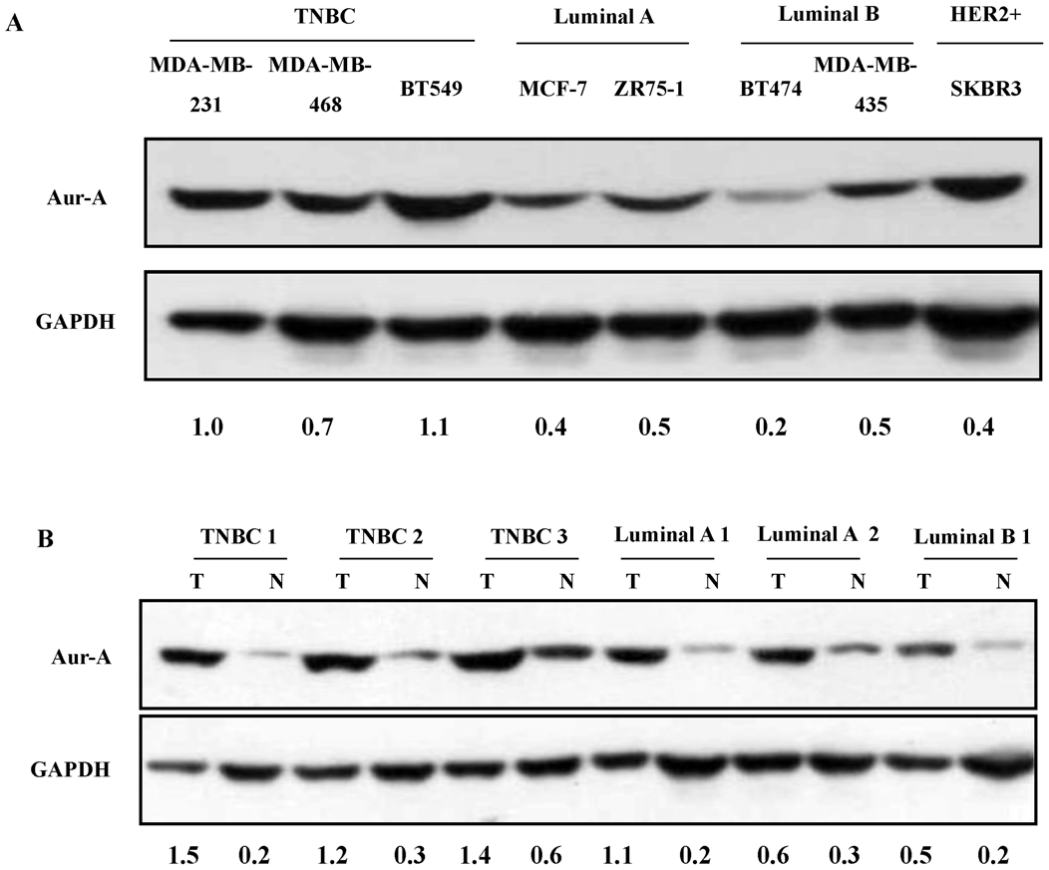
Aur-A was overexpressed in breast cancer cell lines and primary samples. (A) Western blot analysis of Aur-A expression in breast cancer cell lines. (B) Western blot analysis of Aur-A expression in breast cancer samples.

**Figure 5:**
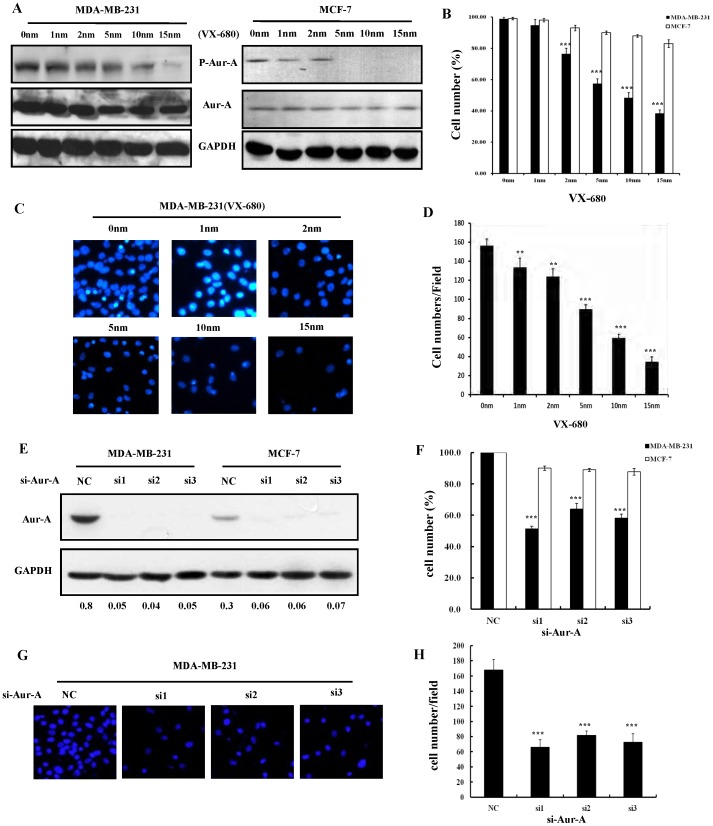
Inhibition of Aur-A kinase inhibited TNBC cell proliferation and reduced cell migration. (A) TNBC cell MDA-MB-231 and non-TNBC cell MCF-7 were incubated with indicated doses of VX-680 (1, 2, 5, 10, and 15 nm), or DMSO for 24 h; Cells were harvested, and subjected to Western blot analysis for the indicated proteins. (B) MDA-MB-231 and MCF-7 cells were exposed to different concentrations of VX-680 (1, 2, 5, 10, and 15 nm) or DMSO for 24 h. Cell survival rates were measured by MTT assay, *P<0.05; **P<0.01; ***P<0.001. (C) and (D) MDA-MB-231 cells were seeded for transwell migration assay in the presence of DMSO or increasing doses of VX-680. After incubation for 24 h, migration rates were quantified by counting the migrated cells in five random fields. Original magnification, 100×. Data summarized three independent experiments. Columns, average cell number; bars, SD. (E) TNBC cell MDA-MB-231 and non-TNBC cell MCF-7 were treated with targeted RNAis or control for 48 h, then cells were harvested, and subjected to Western blot analysis for the indicated proteins. (F) MDA-MB-231 and MCF-7 cells were treated with different RNAis for 48 h. Cell survival rates were then measured by MTT assay. (G) and (H) MDA-MB-231 cells were treated with different RNAis for 24 h, then seeded for transwell migration assay for 24 h, migration rates were quantified by counting the migrated cells in five random fields. Original magnification, 100×.

In order to define the therapeutic role of Aur-A in TNBC, we detected Aur-A expression in various types of breast cancer cell lines (TNBC: MDA-MB-231, MDA-MB-468, BT-549; Luminal A: MCF-7, ZR75-1; Luminal B: BT-474, MDA-MB-435; HER2+: SKBR3) [37] and primary samples. As shown in Figure 4, TNBC cells and samples showed elevated expression of Aur-A compared to non-TNBC cells and tissues. Importantly, a small Aur-A kinase inhibitor VX-680 [16], [20], effectively reducing the Aur-A kinase activity in a dose-dependent manner (Figure 5A; Figure S3A), significantly inhibited cell proliferation (Figure 5B, Figure S3) and reduced cell migration (Figure 5C, D; Figure S3C, D) in TNBC cells (MDA-MB-231, MDA-MB-468) compared to non-TNBC cells (MCF-7, MDA-MB-435). Consistently, Aur-A inhibition with targeted RNAi further demonstrated that cell proliferation and migration of TNBC are uniquely dependent on Aur-A kinase (Figure 5E–H; Figure S3E–H).

## Discussion

As the most aggressive subtype of breast cancer, TNBC occurs in approximately 20∼25% of all patients with breast cancer, associating with an unfavorable prognosis [7], [38], [39]. The risk of recurrence and death is significantly higher for TNBC mainly within the first 3 years of follow-up, and the risk of recurrence decreased thereafter [36], [40]. In the present study, we detected Aur-A expression in TNBC and found that Aur-A was positively associated with the recurrence rate of TNBC (Table 1). TNBC patients with Aur-A high expression displayed an early recurrence within the first 3 years of follow-up, whereas patients with Aur-A low expression showed a relatively constant risk of recurrence during the entire follow-up period (Table 2). Therefore, we suggest that TNBC patients with Aur-A high expression should be followed-up more frequently particularly within the first 3 years to be on guard against any early recurrence and progression.

Recently, the proliferation index Ki-67 is used to further classification of TNBC with different response and prognosis [10]. TNBC is further divided into aggressive (basal like) and less aggressive (non-basal like) subgroups according to the expression status of CK5/6 and EGFR [12]–[14], [41]. Additionally, the basal-like subtype of TNBC was positively correlated with the expression of three biological biomarkers, including c-kit, Ki67, and Aur-A, which are useful to classify TNBC into at least two subtypes [41]. However, they did not identify that Aur-A and Ki-67 are independent prognostic factors for the basal-like subtype of TNBC, which may be partly resulted from the limited sample size (48 for TNBC; 22 for the basal-like subtype) [41]. Herein, we recruited a larger cohort of TNBC patients (n = 122) to investigate the prognostic significance of Aur-A and Ki-67 in TNBC, and determine whether Aur-A promises a therapeutic target for TNBC therapy. Our results showed that Aur-A and Ki-67 were concurrently elevated in TNBC tissues (Figure 1; Figure S1 and S2). Overexpression of Aur-A, associated with high expression of Ki-67, predicted inferior OS and PFS in TNBC (Figure 2; Figure 3), emerging as an adverse independent prognostic factor for TNBC (Table 3, 4). In addition, Ki-67 was found to be a significant independent prognostic factor for OS (Table 3), but not for PFS (Table 4). Taken together, our findings in this study provided novel evidence that Aur-A might be used to classify TNBC patients into two different subgroups with different progression and prognosis.

As the underlying mechanism of Aur-A involving in cancer progression and prognosis, it remains complicated and varies in various types of human cancers. Previously, we found that Aur-A promoted epithelial–mesenchymal transition and invasion in nasopharyngeal carcinoma (NPC) mediated by mitogen-activated protein kinase (MAPK) phosphorylation [31], and increased LSCC cell growth and migration mediated by activation of Akt1 [20]. More recently, we further demonstrated that Aur-A overexpression enhanced breast cancer cell migration by activating the cofilin-F-actin pathway [27]. In the present study, our results showed that Aur-A was positively associated with the proliferation marker Ki-67 (Table 1), and overepxression of Aur-A, associating with high Ki-67, predicted an inferior prognosis of TNBC (Figure 3). Combined with the previous results [20], [27], [31], our findings further supported that an early recurrence and a poor prognosis pattern of TNBC patients with Aur-A high expression might be ascribed to the high proliferation- and metastasis-promoting function of Aur-A. A rational therapeutic strategy, therefore, is needed to target the Aur-A kinases in TNBC.

In our previous studies, VX-680, a selective Aurora kinase inhibitor, was demonstrated to effectively suppress cell growth and migration and potently sensitized cancer cells to therapy, leading to significant cell death in LSCC [20], breast cancer [27], NPC [31], and acute myeloid leukemia (AML) [42]. Here, we found that Aur-A was high expressed in TNBC cell lines and tumor samples, and inhibition of Aur-A kinase by VX-680 or RNAi can efficiently inhibit cell proliferation and decrease cell migration in TNBC cells (Figure 5, Figure S3), suggesting that inhibition of Aur-A kinase may be a potential therapeutic strategy for TNBC treatment. Encouragingly, a phase 1/2 study shows that 3 patients with T315I phenotype–refractory CML (Chronic Myeloid Leukemia) or Ph-positive ALL (Acute Lymphocytic Leukemia) have achieved clinical responses to doses of MK-04547 (VX-680) that are not associated with significant extramedullary toxicity [43]. More recently, a phase 1 study reported that MK-0457 (VX-680) was fairly well tolerated in the scheduled doses in a population of 27 patients with advanced solid tumors (including 1 breast cancer patients), and almost half of the patients attained stable disease [44]. Thus, we suggest that agents targeting Aur-A (e.g. VX-680) with or without concurrent use of adjuvant chemotherapy for TNBC patients with Aur-A high expression may be tolerated and feasible to decrease the likelihood of disease recurrence and poor prognosis.

In conclusion, the findings reported here provide several new insights to the understanding of TNBC as following: (a) Aur-A is useful to identify TNBC patients that are at high risk of early recurrence and progression. (b) Overexpression of Aur-A, associated with high expression of Ki-67, predicts a shorter survival of TNBC, serving as a novel independent prognostic biological marker for TNBC. (c) Aur-A is demonstrated as a potential therapeutic target for TNBC and inhibition of Aur-A kinase is a promising regimen for cancer therapy in TNBC patients with Aur-A high expression.

## Supporting Information

Figure S1 **Whole picture of TMA with Aur-A staining.**
(TIF)

Figure S2 **Immunohistochemistry analysis of Aur-A and Ki-67 expression in TNBC tissues.** (A1) Ki-67 low expression and (A2) Aur-A high expression was shown in a TNBC patient sample (100×). (B1) Ki-67 low expression and (B2) Aur-A low expression was shown in a TNBC patient sample (100×). (C1) Ki-67 high expression and (C2) Aur-A high expression was shown in a TNBC patient sample (100×). (D1) Ki-67 high expression and (D2) Aur-A low expression was shown in another sample (100×). (A1 ’), (A2 ’), (B1 ’), (B2 ’), (C1 ’), (C2 ’), (D1 ’), (D2 ’) demonstrated the higher magnification (200×) from the area of the box in (A1), (A2), (B1), (B2), (C1), (C2), (D1), (D2) respectively.
(TIF)

Figure S3 **Inhibition of Aur-A kinase inhibited TNBC cell proliferation and reduced cell migration.** (A) TNBC cell MDA-MB-468 and non-TNBC cell MDA-MB-435 were incubated with indicated doses of VX-680 (1, 2, 5, 10, and 15 nm), or DMSO for 24 h; Cells were harvested, and subjected to Western blot analysis for the indicated proteins. (B) MDA-MB-468 and MDA-MB-435 cells were exposed to different concentrations of VX-680 (1, 2, 5, 10, and 15 nm) or DMSO for 24 h. Cell survival rates were measured by MTT assay, *P<0.05; **P<0.01; ***P<0.001. (C) and (D) MDA-MB-468 cells were seeded for transwell migration assay in the presence of DMSO or increasing doses of VX-680. After incubation for 24 h, migration rates were quantified by counting the migrated cells in five random fields. Original magnification, 100×. Data summarized three independent experiments. Columns, average cell number; bars, SD. (E) TNBC cell MDA-MB-468 and non-TNBC cell MDA-MB-435 were treated with targeted RNAis or control for 48 h, then cells were harvested, and subjected to Western blot analysis for the indicated proteins. (F) MDA-MB-231 and MCF-7 cells were treated with different RNAis for 48 h. Cell survival rates were then measured by MTT assay. (G) and (H) MDA-MB-468 cells were treated with different RNAis for 24 h, then seeded for transwell migration assay for 24 h, migration rates were quantified by counting the migrated cells in five random fields. Original magnification, 100×.
(TIF)
