# Supplementary figures and images for: Aurora-A Identifies Early Recurrence and Poor Prognosis and Promises a Potential Therapeutic Target in Triple Negative Breast Cancer
Source: PLoS One. 2013 Feb 20;8(2):e56919. doi: 10.1371/journal.pone.0056919 (PMC3577665; doi:10.1371/journal.pone.0056919)

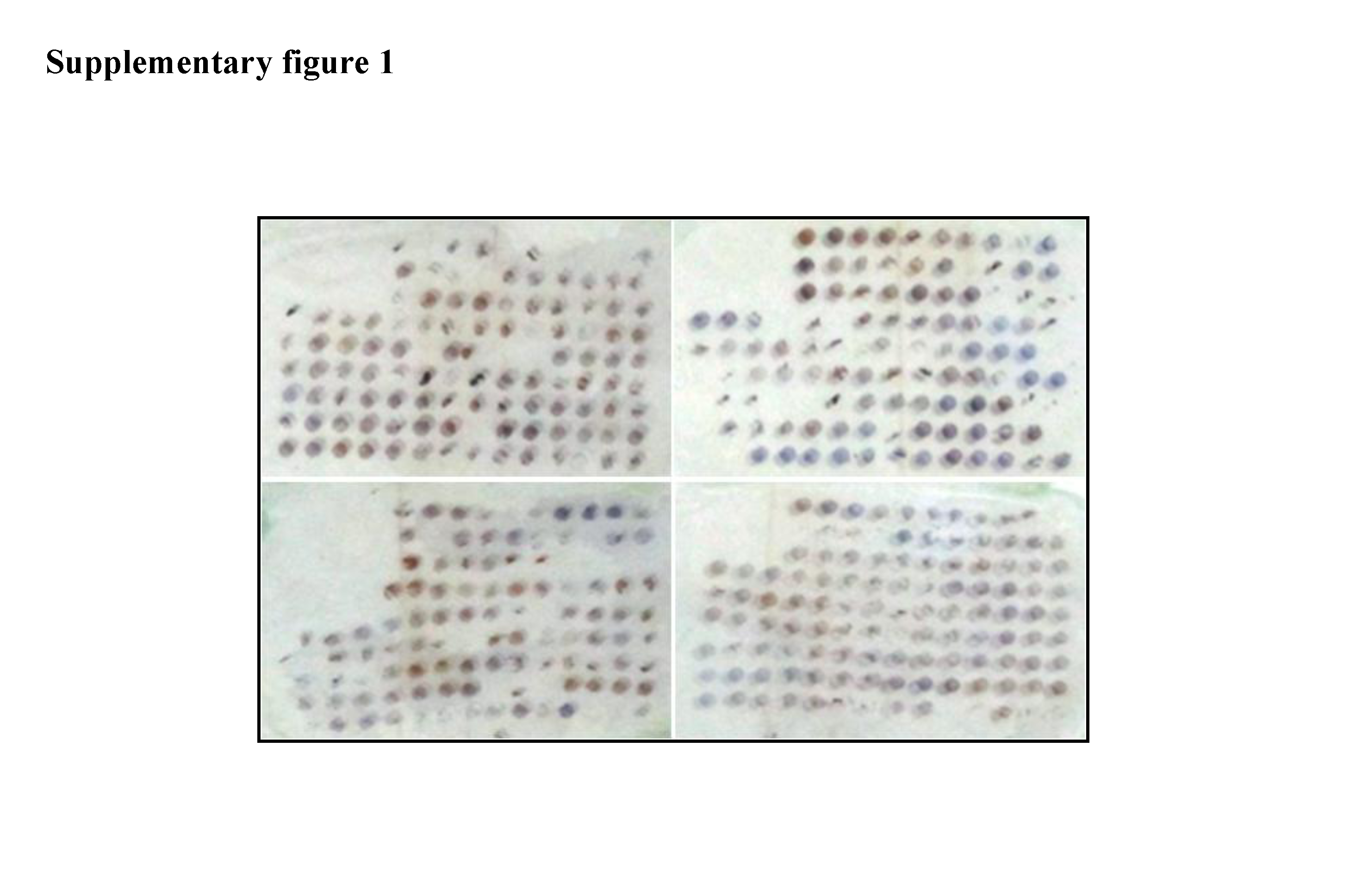

Supplement: Figure S1 — Whole picture of TMA with Aur-A staining. (TIF) [file pone.0056919.s001.tif]

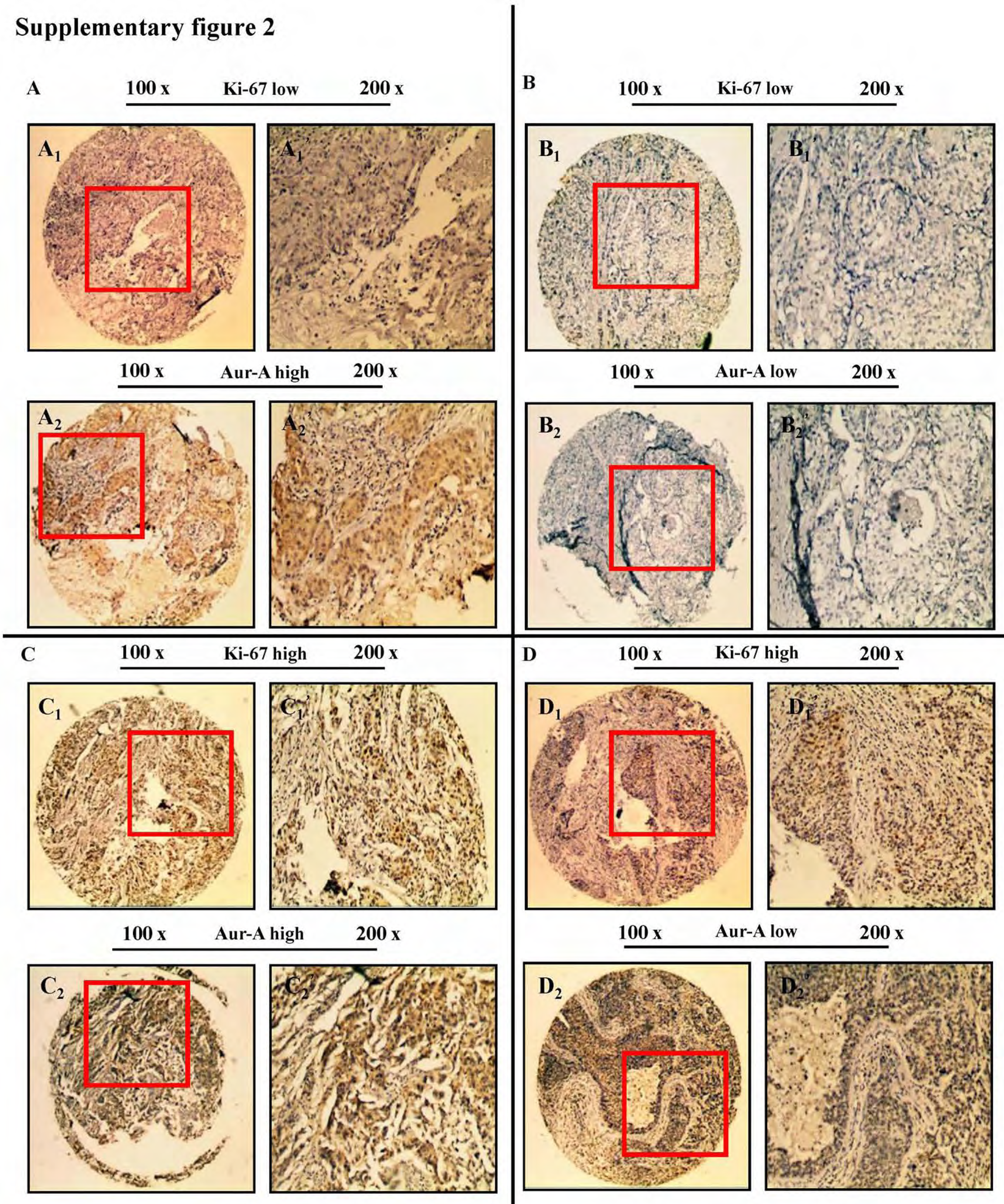

Supplement: Figure S2 — Immunohistochemistry analysis of Aur-A and Ki-67 expression in TNBC tissues. (A1) Ki-67 low expression and (A2) Aur-A high expression was shown in a TNBC patient sample (100×). (B1) Ki-67 low expression and (B2) Aur-A low expression was shown in a TNBC patient sample (100×). (C1) Ki-67 high expression and (C2) Aur-A high expression was shown in a TNBC patient sample (100×). (D1) Ki-67 high expression and (D2) Aur-A low expression was shown in another sample (100×). (A1 ’), (A2 ’), (B1 ’), (B2 ’), (C1 ’), (C2 ’), (D1 ’), (D2 ’) demonstrated the higher magnification (200×) from the area of the box in (A1), (A2), (B1), (B2), (C1), (C2), (D1), (D2) respectively. (TIF) [file pone.0056919.s002.tif]

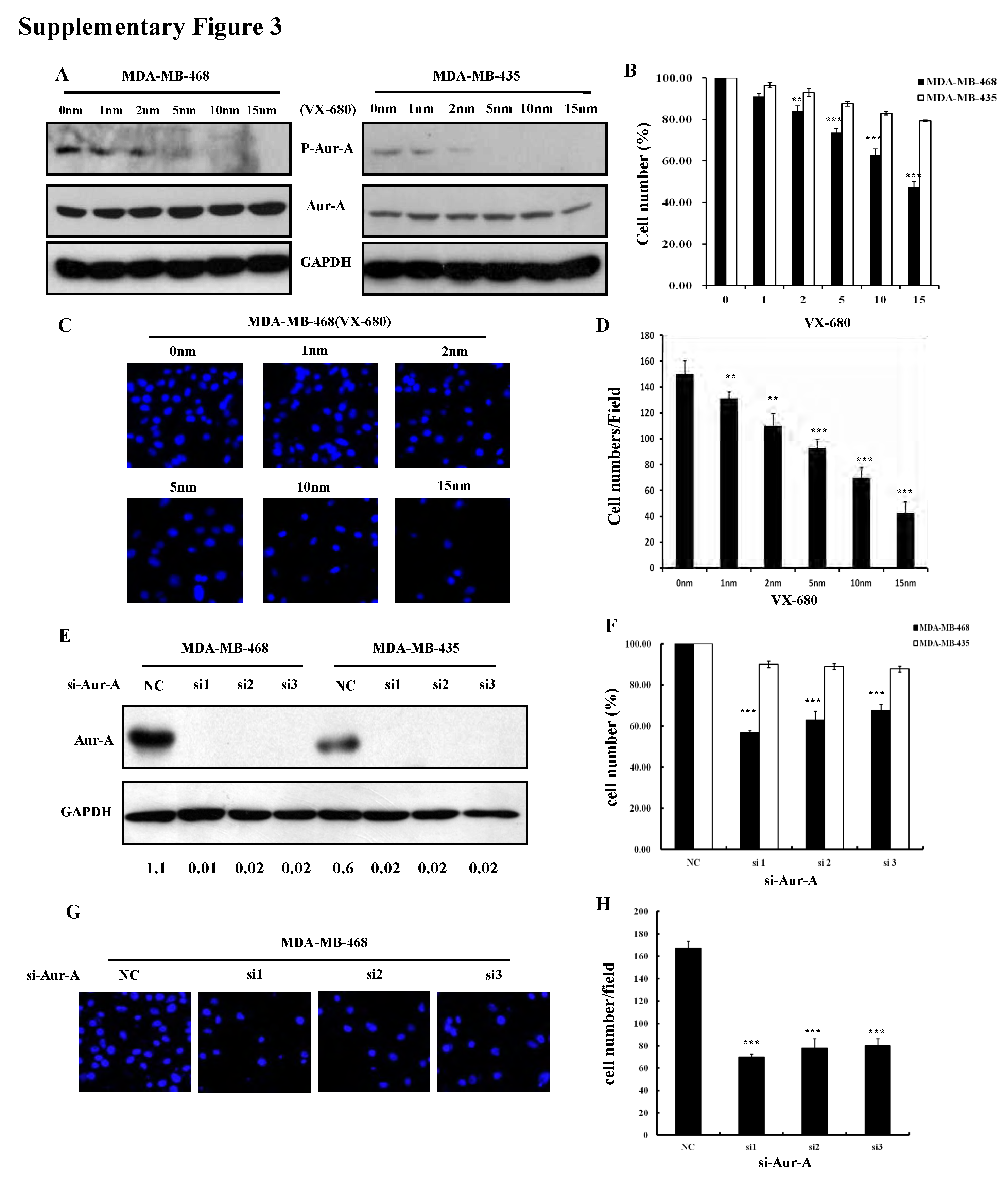

Supplement: Figure S3 — Inhibition of Aur-A kinase inhibited TNBC cell proliferation and reduced cell migration. (A) TNBC cell MDA-MB-468 and non-TNBC cell MDA-MB-435 were incubated with indicated doses of VX-680 (1, 2, 5, 10, and 15 nm), or DMSO for 24 h; Cells were harvested, and subjected to Western blot analysis for the indicated proteins. (B) MDA-MB-468 and MDA-MB-435 cells were exposed to different concentrations of VX-680 (1, 2, 5, 10, and 15 nm) or DMSO for 24 h. Cell survival rates were measured by MTT assay, *P<0.05; **P<0.01; ***P<0.001. (C) and (D) MDA-MB-468 cells were seeded for transwell migration assay in the presence of DMSO or increasing doses of VX-680. After incubation for 24 h, migration rates were quantified by counting the migrated cells in five random fields. Original magnification, 100×. Data summarized three independent experiments. Columns, average cell number; bars, SD. (E) TNBC cell MDA-MB-468 and non-TNBC cell MDA-MB-435 were treated with targeted RNAis or control for 48 h, then cells were harvested, and subjected to Western blot analysis for the indicated proteins. (F) MDA-MB-231 and MCF-7 cells were treated with different RNAis for 48 h. Cell survival rates were then measured by MTT assay. (G) and (H) MDA-MB-468 cells were treated with different RNAis for 24 h, then seeded for transwell migration assay for 24 h, migration rates were quantified by counting the migrated cells in five random fields. Original magnification, 100×. (TIF) [file pone.0056919.s003.tif]
